# A multicenter prospective registry of Borden type I dural arteriovenous fistula: results of a 3-year follow-up study

**DOI:** 10.1007/s00234-021-02752-5

**Published:** 2021-10-10

**Authors:** Hidehisa Nishi, Hiroyuki Ikeda, Akira Ishii, Takayuki Kikuchi, Ichiro Nakahara, Tsuyoshi Ohta, Nobuyuki Sakai, Hirotoshi Imamura, Jun C. Takahashi, Tetsu Satow, Tomohisa Okada, Susumu Miyamoto

**Affiliations:** 1 Department of Neurosurgery, Kyoto University Graduate School of Medicine, 54 Kawahara-cho, Shogoin, Sakyo-ku, Kyoto, 606-8507 Japan; 2 Department of Neurosurgery, National Hospital Organization Kyoto Medical Center, Kyoto, Japan; 3 Department of Neurosurgery, Kurashiki Central Hospital, Kurashiki, Japan; 4 Department of Neurosurgery, Kokura Memorial Hospital, Kitakyushu, Japan; 5 Department of Comprehensive Strokology, Fujita Health University School of Medicine, Toyoake, Japan; 6 Department of Neurosurgery, National Cerebral and Cardiovascular Center, Suita, Japan; 7 Department of Neurosurgery, Kobe City Medical Center General Hospital, Kobe, Japan; 8 Department of Neurosurgery, Kindai University Hospital, Osaka-Sayama, Japan; 9 Department of Radiology, Kyoto University Graduate School of Medicine, Kyoto, Japan

**Keywords:** Dural arteriovenous fistula, Natural history, Borden type I

## Abstract

**Purpose:**

Although intracranial dural arteriovenous fistula (DAVF) without retrograde leptomeningeal venous drainage (Borden type I) is reported to have a benign nature, no study has prospectively determined its clinical course. Here, we report a 3-year prospective observational study of Borden type I DAVF.

**Methods:**

From April 2013 to March 2016, consecutive patients with DAVF were screened at 13 study institutions. We collected data on baseline characteristics, clinical symptoms, angiography, and neuroimaging. Patients with Borden type I DAVF received conservative care while palliative intervention was considered when the neurological symptoms were intolerable, and were followed at 6, 12, 24, and 36 months after inclusion.

**Results:**

During the study period, 110 patients with intracranial DAVF were screened and 28 patients with Borden type I DAVF were prospectively followed. None of the patients had conversion to higher type of Borden classification or intracranial hemorrhage during follow-up. Five patients showed spontaneous improvement or disappearance of neurological symptoms (5/28, 17.9%), and 5 patients showed a spontaneous decrease or disappearance of shunt flow on imaging during follow-up (5/28, 17.9%). Stenosis or occlusion of the draining sinuses on initial angiography was significantly associated with shunt flow reduction during follow-up (80.0% vs 21.7%, *p* = 0.02).

**Conclusion:**

In this 3-year prospective study, patients with Borden type I DAVF showed benign clinical course; none of these patients experienced conversion to higher type of Borden classification or intracranial hemorrhage. The restrictive changes of the draining sinuses at initial diagnosis might be an imaging biomarker for future shunt flow reduction.

## Introduction

Several studies have explored the natural history of intracranial dural arteriovenous fistula (DAVF) without retrograde leptomeningeal venous drainage (RLVD), classified as Borden type I [1–5]. Most of these previous reports had indicated that the natural history of DAVF without RLVD is a benign type. Reported rates of intracranial hemorrhage, newly acquired nonhemorrhagic neurological deficit (NHND), and mortality have been extremely low: 0–1%. In addition, spontaneous occlusion of the disease has been shown to occur in 3–13% of patients. However, 0–8% of patients have displayed an angiographical conversion to RLVD.

The results of Borden type I DAVF noted above were based on retrospective case series with various observation periods, which limits the reliability of the results. Also, the more detailed time-dependent change in the disease will not become clear without prospective observation with scheduled follow-up.

Here, we report a 3-year prospective observational study of DAVF without RLVD. The purpose of the study was to reveal the detailed clinical course of Borden type I DAVF under a standard conservative treatment strategy.

## Materials and methods

### Patients

From April 2013 to March 2016, consecutive patients with intracranial DAVF were screened at 13 study institutions in Japan. Patient information (age, sex, neurological symptoms, and medical history) and imaging data (cerebral angiography and brain magnetic resonance imaging) were submitted to the central office at the time of screening. All the diagnoses and classifications of Borden type were confirmed by 2 independent neurointerventionalists. When a disagreement occurred, a third rater was assigned and the disagreement was settled among the 3 raters. The inclusion criterion was intracranial DAVF classified as Borden type I by digital subtraction angiography. Exclusion criteria were arteriovenous shunts caused by trauma, cerebral aneurysm rupture, and arteriovenous shunts related to craniotomy. Patients for whom outpatient follow-up were not possible were also excluded.

Given the completely observational nature of the study, consent to participate in this study was obtained with an opt-out approach. The study was approved by the ethics committees of each participating institution.

### Study endpoint

Patients were followed up at 6, 12, 24, and 36 months after study inclusion. At the follow-up, patients were assessed for any stroke event, neurological symptom changes, and their activities of daily living status by modified Rankin scale. In addition, all patients were scheduled to undergo brain magnetic resonance imaging (MRI) at each follow-up. When a Borden classification type conversion was suspected by symptom worsening or changes in MRI findings, a cerebral angiography was considered (decision to perform follow-up cerebral angiography was at the discretion of the treating physician). Palliative intervention was not omitted to alleviate patient symptoms, especially when the symptoms were intolerable.

The primary endpoint was death or any type of stroke during the study period. The secondary endpoints were morbidity related to the disease, the status of symptoms related to the disease, any changes related to the disease on brain MRI, conversion to a higher Borden classification type, and any intervention toward intracranial DAVF. NHND was defined as neurological symptoms, including seizure, dementia, parkinsonism, focal cortical deficits, cranial nerve palsies, trigeminal neuralgia, and cerebellar dysfunction. Orbital phenomena were not included as NHND, according to previous reports [1].

### The assessment of brain magnetic resonance imaging findings

All the patients received fluid-attenuated inversion recovery, T2 star or susceptibility-weighted imaging, and time-of-flight magnetic resonance angiography (MRA); other sequences were left to the standard protocol at each institution. All the neuroimaging data were collected at the central office and assessed by 2 independent raters: a neuroradiologist (TO) and a neurointerventionalist (HN), each with > 10 years of experience.

The presence of new ischemic or hemorrhagic lesions, an increase or decrease of shunt flow, and the status of venous congestion were assessed. Disagreements were settled by consensus between the 2 raters.

### Statistical analysis

Continuous variables were expressed as mean ± standard deviation or median with interquartile range, depending on the distribution of the variables. For comparing baseline variables and outcomes in the subgroup analyses, the Mann–Whitney U test was used for continuous variables, and the Fisher exact test was used for categorical variables. For the follow-up data, a Kaplan–Meier analysis was performed to analyze the event rate of each endpoint. Two-sided p values of < 0.05 was considered to indicate statistical significance. We performed the statistical analyses with the R statistical software (version 4.0.0).

## Results

### Data availability

The data that support the findings of this study are available, upon reasonable request, from the corresponding author.

### Baseline characteristics

During the study period, 110 patients with intracranial DAVF were screened, with 40 patients classified as Borden without RLVD (type I), 27 patients classified as Borden type II, and 43 patients classified as Borden type III.

**Fig. 1:**
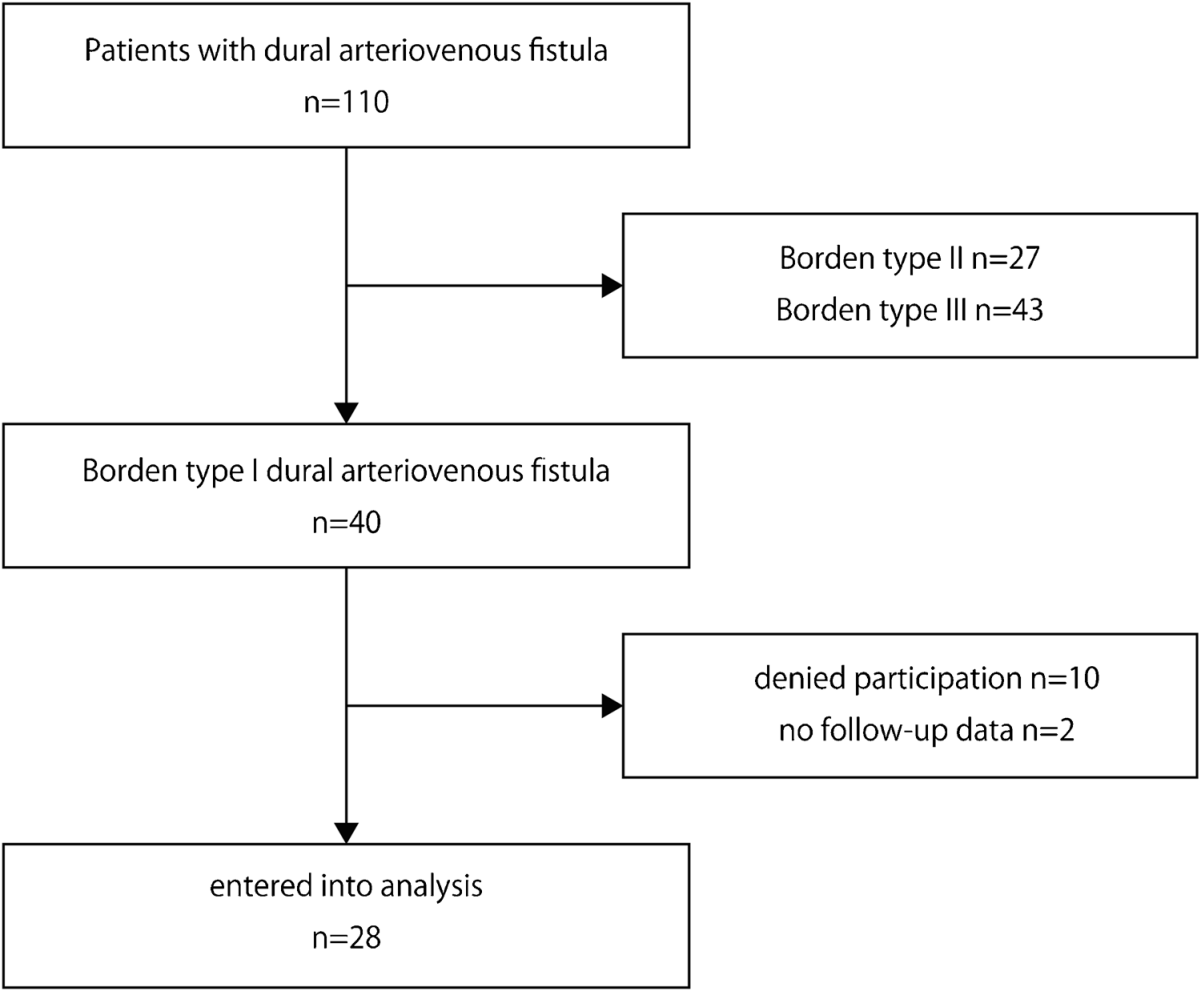
Flow diagram of the patients included in this study. Among 110 patients with intracranial DAVF, 40 patients had Borden type I DAVF. At inclusion, 10 patients denied participation or were unable to participate in the outpatient follow-up. After inclusion, 2 patients dropped out and had no follow-up data. Ultimately, the data of 28 patients were analyzed

Among the 40 patients classified as Borden I, 30 patients met the inclusion criteria and agreed to participation in the study. During follow-up, 5 patients dropped out and 25 patients (25/30, 83.3%) completed the follow-up. The mean follow-up period was 33.0 months (77.8 patient-years). Ultimately, 28 patients who were followed up more than 6 months were included in the analysis (Fig. 1).

All the patients are listed in Tables 1 and 2. The mean age was 63.6 ± 14.9 years, with 15/28 (53.6%) female. The most common disease location was the transverse-sigmoid sinus (17/28, 60.7%), followed by the anterior condylar confluence (5/29, 17.9%). Tinnitus was the most common symptom (17/28, 60.7%), followed by headache (5/28, 17.9%) and asymptomatic patients (5/28, 17.9%). Two patients had an NHND, which were higher-order dysfunction and epilepsy (2/28, 7.1%). Seventeen patients were classified as Cognard type I (60.7%) and 11 patients as type IIa (30.3%).

**Table 1:** The patients with spontaneous shunt flow reduction or obliteration during follow-up

Case no	Age (years), sex	Side, location	Feeding artery	Draining vein	Stenosis or occlusion of the draining veins	Initial symptom	Spontaneous change in symptoms	Spontaneous change in MRI findings	Intervention	Clinical outcome (modified Rankin scale score)
9	42, F	Rt. TS/SS	MHT, MMA, APhA	Transverse sinus	Yes	Tinnitus, headache	Yes, disappear	Yes, Shut flow reduction	No	0
15	42, F	Lt. TS/SS	MHT, MMA, OA	Sigmoid sinus, transverse sinus	Yes	Tinnitus	Yes, disappear	Yes, flow disappearance	No	0
19	83, M	Lt. TS/SS	OA, APhA	Sigmoid sinus	Yes	None	No	Yes, flow reduction	No	0
21	79, F	Lt. TS/SS	OA, MMA, APhA	Sigmoid sinus, transverse sinus	Yes	Tinnitus	Yes, transient worsening (6 M), then disappeared (24 M)	Yes, flow reduction	No	0
22	72, F	Rt. TS/SS	OA, MMA, APhA	Sigmoid sinus	No	Eyeache	Yes, disappear	Yes, flow disappearance	No	0

*TS/SS* transvers sinus/sigmoid sinus, *MMA* middle meningeal artery, *AMA* accessory meningeal artery, *APhA* ascending pharyngeal artery, *OA* occipital artery, *VA* vertebral artery, *AOFR* artery of foramen rotundum, *MHT* meningohypothyseal trunk, *TAE* transarterial embolization, *TVE* transvenous embolization

**Table 2:** The patients without spontaneous shunt flow reduction or obliteration during follow-up

Case no	Age (years), sex	Side, location	Feeding artery	Draining vein	Stenosis or occlusion of the draining veins	Initial symptom	Spontaneous change in symptoms	Spontaneous change in MRI findings	Intervention	Clinical outcome (modified Rankin scale score)
1	60, F	Lt. TS/SS	OA, MMA, APhA, VA	Internal jugular vein, Inferior petrosal sinus, transverse sinus	No	Tinnitus	No	No	No	1
2	68, M	Lt. ACC	APhA, VA	Internal jugular vein	No	Tinnitus	No	No	No	1
3	45, M	Rt. TS/SS	OA	Sigmoid sinus	Yes	None	Yes, new onset of tinnitus	No	No	0
4	71, M	Rt. ACC	APhA, OA	Internal jugular vein, sigmoid sinus	No	Tinnitus	Yes, worsening	No	No	1
5	78, F	Lt. TS/SS	OA, MMA, PAA	Sigmoid sinus, transverse sinus	No	Tinnitus	No	No	Yes (TAE)	0
6	69, F	Lt. CS	MHT, inferolateral trunk	Inferior petrosal sinus	No	Headache, eye movement disorder	No	No	Yes (TVE)	0
7	65, F	Lt. TS/SS	OA, MMA, PAA	Sigmoid sinus	Yes	Tinnitus	No	No	Yes (TAE)	0
8	74, M	Confluence	OA, VA	Transverse sinus	Yes	Higher brain dysfunction	Yes, worsening	No	Yes (TAE)	4
10	47, F	Rt. TS/SS	OA, MMA	Sigmoid sinus	No	Tinnitus	No	No	No	1
11	59, M	Confluence	OA, MMA	Occipital sinus, superior sagittal sinus, straight sinus	Yes	Tinnitus	No	No	Yes (TAE)	1
12	86, M	Lt. TS/SS	OA, MMA, VA	Sigmoid sinus	No	None	No	No	No	0
13	32, F	Rt. ACC	OA, VA	Internal vertebral venous plexus, cervical venous plexus	No	Tinnitus	No	No	Yes (TAE)	0
14	63, F	Lt. TS/SS	OA, MMA, STA	Sigmoid sinus	No	Tinnitus, seizure	No	No	No	0
16	42, F	SSS	MMA, Ophthalmic artery (recurrent meningeal artery)	Superior sagittal sinus	No	Headache	No	No	No	1
17	58, M	Lt. ACC	OA, APhA, VA	Internal jugular vein, inferior petrosal sinus	No	Tinnitus	No	No	No	1
18	62, M	Rt. TS/SS	OA, APhA	Sigmoid sinus, transverse sinus	No	None	Yes, new onset of tinnitus	No	No	1
20	74, M	Rt. TS/SS	OA, MMA, STA, deep cervical artery	Transverse sinus, sigmoid sinus	No	Tinnitus	No	No	Yes (TAE/TVE)	2
23	77, F	Lt. TS/SS	OA, STA, PAA	Sigmoid sinus	Yes	Tinnitus	No	No	Yes (TAE)	0
24	40, M	Rt. TS/SS	OA, APhA	Sigmoid sinus	No	Headache	Yes, headache disappear (6 M), new onset of tinnitus (36 M)	No	No	1
25	78, M	Rt. ACC	APhA, VA, artery of the pterygoid canal	Internal jugular vein	No	Tinnitus	No	No	No	1
26	67, F	Rt. CS	MHT, APhA	Inferior petrosal sinus, superior petrosal sinus	No	Eye movement disorder	No	No	Yes (TAE/TVE, radiosurgery)	0
27	74, M	Rt. CS	MHT, artery of the pterygoid canal, artery of foramen rotundum, APhA	Inferior petrosal sinus	No	None	No	No	No	0
28	76, F	Lt. TS/SS	MMA, OA	Sigmoid sinus, transverse sinus	No	Tinnitus	No	No	Yes (TAE/TVE)	1

*TS/SS* transvers sinus/sigmoid sinus, *ACC* anterior condylar canal, *CS* cavernous sinus, *SSS* superior sagittal sinus, *MMA* middle meningeal artery, *AMA* accessory meningeal artery, *APhA* ascending pharyngeal artery, *OA* occipital artery, *VA* vertebral artery, *STA* superficial temporal artery, *PAA* posterior auricular artery, *MHT* meningohypothyseal trunk, *TAE* transarterial embolization, *TVE* transvenous embolization

### Primary endpoint: any death or stroke event

**Fig. 2:**
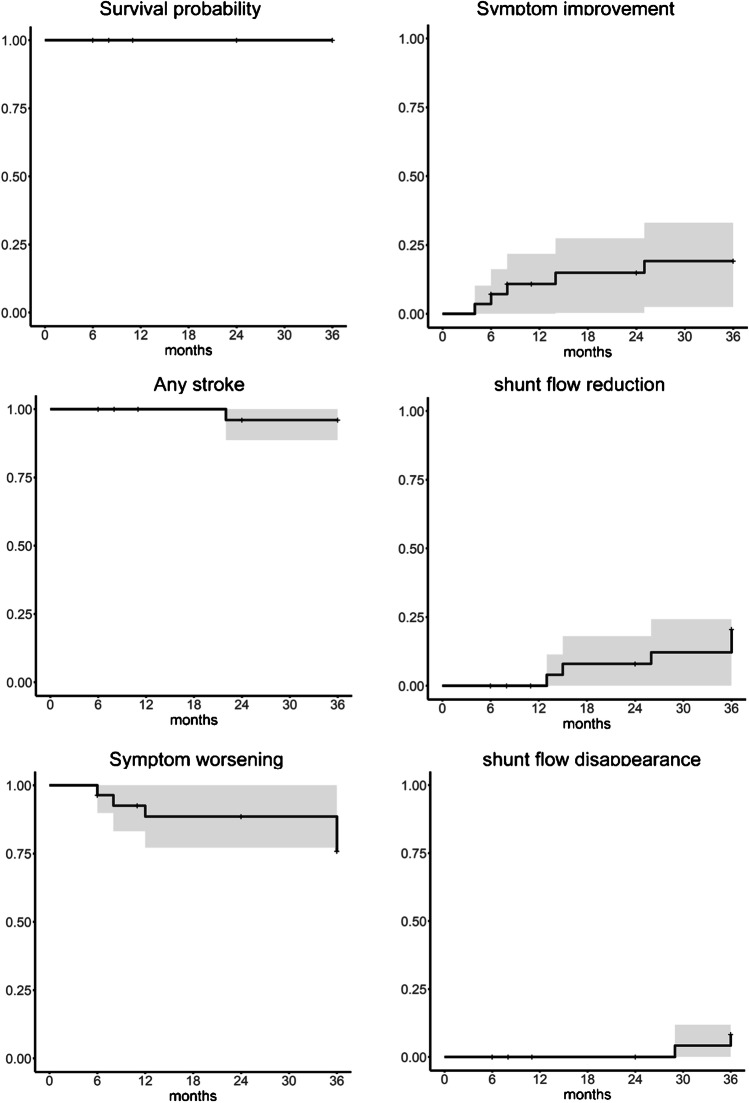
Kaplan–Meier curve for each endpoint (death, any stroke, spontaneous symptom improvement, spontaneous symptom worsening, spontaneous shunt flow reduction, and spontaneous shunt flow disappearance). Each event curve is shown with a 95% confidence interval (gray area)

There were no deaths (0/28, 0.0%) and no intracranial hemorrhages (0/28, 0.0%) during follow-up. Otherwise, 1 patient had a lacunar infarction at the right corona radiata after 2 years from inclusion, which was not considered to be related to the DAVF (1/28, 3.8%) (Table 2) (Fig. 2).

### Neurological symptom changes and therapeutic interventions

During follow-up, nearly 40% of the patients displayed a change in symptoms. Five patients showed spontaneous symptom improvement during follow-up (5/28, 17.9%). On the other hand, 6 patients showed symptom worsening (6/28, 21.4%); brain MRI was performed in all six cases, and three patients were further evaluated with angiography. None of the patients displayed Borden type conversion with DSA; one patient displayed increase in the shunt flow, while the other two patients displayed no apparent changes in angioarchitecture. One case of progression to higher brain dysfunction led to an increase in the modified Rankin scale from 1 to 4 (the morbidity case; 1/28, 3.8%).

To alleviate the neurological symptoms, neurointerventional procedures were performed in 10 patients for 12 sessions (10/28, 35.7%), and radiosurgery was performed in 1 patient once (1/28, 3.8%). After these interventions, 7 patients reached partial occlusion and 3 achieved immediate complete obliteration.

The estimated rate of symptom improvement was 19.3% per 3 years, and symptom worsening was 20.0% per 3 years (Fig. 2).

### The findings on magnetic resonance imaging and cerebral angiography

All untreated patients completed the scheduled follow-up MRI until 36 months, except one patient with asymptomatic transverse-sigmoid sinus DAVF who denied MRI at the 36-month follow-up. There was no sign of intracranial hemorrhage or worsening of cortical venous congestion on T2-star-weighted images (0/28, 0.0%). During follow-up, 1 patient showed new cerebral infarction at the right corona radiata, which was mentioned above (1/28, 3.8%).

**Fig. 3:**
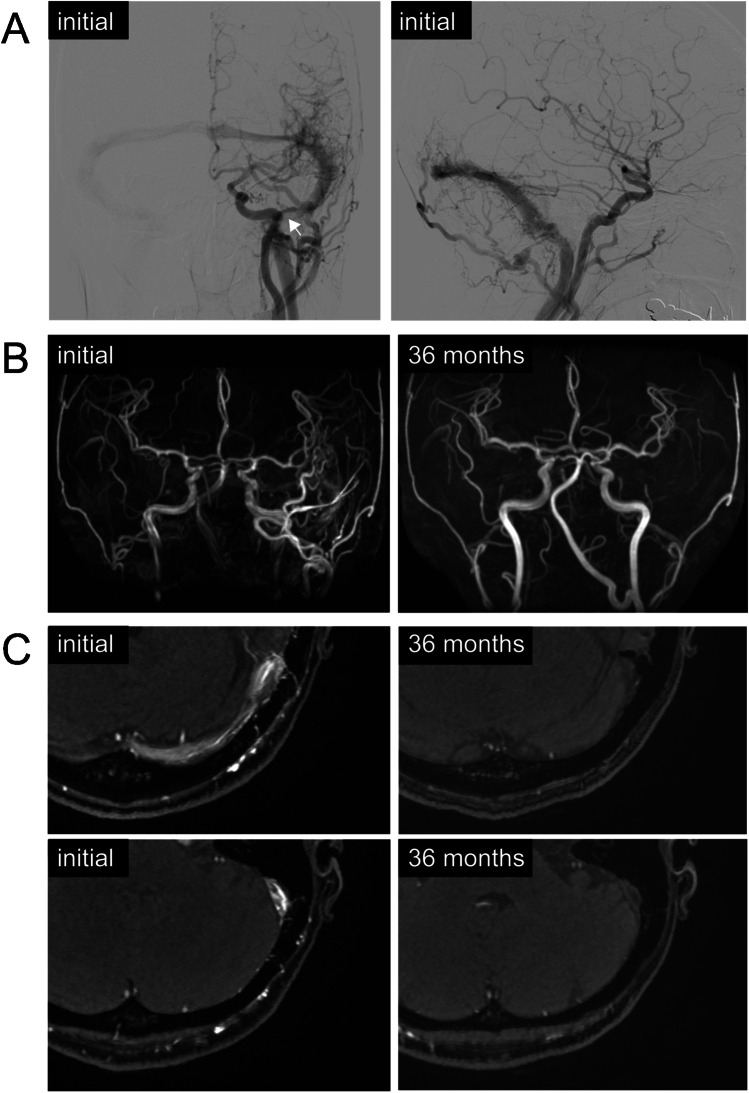
Representative case with spontaneous shunt flow reduction on magnetic resonance angiography: an adult female with dural arteriovenous fistula at the left transverse-sigmoid sinus. Angiography displays multiple feeders from internal and external carotid arteries (**A**). The stenosis of the left sigmoid sinus was present (arrow). The patient had tinnitus, which resolved at 6 months of follow-up. On magnetic resonance angiography, the shunt flow disappeared after 24 months of follow-up (**B**, **C**)

On MRA, 5 patients showed spontaneous shunt-flow reduction (5/28, 17.9%). All 5 cases were located at the transverse-sigmoid sinus, and 4 of them were preceded by spontaneous symptom improvement by 6–12 months, except for 1 asymptomatic case. Among these 5 patients, shunt flow disappeared in 2 patients on MRA (2/28, 7.1%). Clinical symptoms also disappeared in both cases. Otherwise, none of these findings was confirmed by angiography. The representative case is shown in Fig. 3.

The estimated annual rate of shunt flow reduction on MRA was 16.6% per 3 year, and shunt disappearance on MRA was 8.5% per 3 years (Fig. 2).

Follow-up cerebral angiography was acquired only in 12 patients (42.9%), which includes the evaluation on the timing of the intervention. As mentioned in the above section, we found increased shunt blood flow in one case with symptom exacerbation, while no type change of Borden classification was confirmed in any of the cases (Tables 3 and 4).

**Table 3:** Clinical outcomes

Any stroke	1/28 (3.6%)
Ischemic stroke	1/28 (3.6%)
Hemorrhagic stroke	0/28 (0.0%)
Any spontaneous changes of symptoms	11/28 (39.2%)
Symptom improvement	5/28 (17.9%)
< 1 year after inclusion	3/28 (10.7%)
1—2 year after inclusion	1/28 (3.6%)
2—3 year after inclusion	1/28 (3.6%)
Symptom worsening	6/28 (21.4%)
< 1 year after inclusion	2/28 (7.1%)
1—2 year after inclusion	1/28 (3.6%)
2—3 year after inclusion	3/28 (10.7%)
Morbidity	1/28 (3.6%)
Mortality	0/28 (0.0%)

*MRI* magnetic resonance imaging

**Table 4:** Neuroimaging outcomes

Any spontaneous changes on MRI	7/28 (25.0%)
Decreased shunt flow	5/28 (17.9%)
< 1 year after inclusion	0/28 (0.0%)
1—2 year after inclusion	4/28 (14.3%)
2—3 year after inclusion	1/28 (3.6%)
Disappearance of shunt flow	2/28 (7.1%)
< 1 year after inclusion	0/28 (0.0%)
1—2 year after inclusion	2/28 (7.1%)
2—3 year after inclusion	0/28 (0.0%)
Worsening of venous congestion	0/28 (0.0%)
Type conversion to a higher Borden type classification	0/28 (0.0%)

### Subgroup analysis: comparison between patients with spontaneous shunt flow reduction and the others

The patients were divided into two subgroups according to whether shunt flow was spontaneously reduced or not during follow-up. While no apparent difference in biological backgrounds, such as patient age, sex, and comorbidities were found, the diagnostic subtraction angiography findings at initial diagnosis were different between two groups. The presence of the stenosis or occlusion of the draining sinus was significantly common in the shunt-flow reduction group (80.0% vs 21.7%, *p* = 0.02). The number of feeders was slightly larger in the shunt-flow reduction group, but not statistically significant (7 [3–7] vs 3 [2–4, 6], *p* = 0.10).

## Discussion

This is the first prospectively registered data on the clinical outcomes of the patients with Borden type I DAVF. In this study, we prospectively confirmed that Borden type I DAVF shows a benign clinical course with a conservative treatment strategy, which basically involves observing patients while considering palliative interventions when the symptoms are intolerable.

Nearly 20% of the patients displayed symptom improvement and spontaneous shunt flow reduction, and 7% of the cases displayed shunt flow disappearance. Symptom improvement was significantly associated with shunt flow reduction on MRA. In most cases, symptom improvement preceded the decrease in shunt flow on MRA. Although few reports have examined the relationship between symptom improvement and imaging findings, several reports have documented spontaneous occlusion. Gross et al. reported the natural history of 126 untreated patients with Borden type I DAVF and found that spontaneous obliteration occurred in 3% of the cases [4]. Shah et al. reported the clinical course of 23 patients with Borden type I DAVF, and reported that 1 patient obtained complete obliteration after long-term follow-up (4%) [3]. Kim et al. reported that among 99 patients with Borden type I DAVF, 12 patients displayed spontaneous occlusion (12%) [6]. Although our assessment was based on MRA, the obliteration rate was within the range of the previous reports: 3–12%.

The ability to predict spontaneous obliteration in advance can help improve the treatment strategy for Borden type I DAVF. In this study, the stenotic changes in the affected sinuses at initial diagnosis were more frequently observed in the patients with spontaneous shunt flow reduction during follow-up. This finding might suggest that the restrictive change of draining sinus is a predictor of more dynamic change in angioarchitecture and future shunt flow reduction/obliteration. In cavernous sinus lesions, the chronological and progressive restrictive changes in the affected sinus have been reported to lead to spontaneous occlusion [8, 9]. If these unidirectional changes are common to other lesions, it is not surprising that patients with stenotic changes already observed at the time of initial diagnosis are more prone to shunt occlusion in the future. On the other hand, various mechanisms have been reported to be involved in the spontaneous occlusion of the DAVF, such as thrombosis of the dural sinus, inflammatory changes, intracranial hemorrhage, dilatational changes in the venous sinus structure, and consequent hemodynamic changes [6, 10–12]. Although various interactive mechanisms must be involved in the process of spontaneous obliteration of DAVF, stenosis and occlusion of the affected sinus emerged as a biomarker to predict shunt flow reduction in DAVF in this study. Further validation in a large number of patients is needed.

On the other hand, approximately 20% of patients in our cohort displayed worsening of symptoms, but none displayed conversion to a higher type of Borden classification. The reported rate of conversion to a higher type was relatively low: 0–8%, and it is not inconsistent that there was no case of angiographically malignant conversion, given that there were only 28 cases in the entire study population [1–3, 6]. Longer follow-up is recommended, given that the type conversion may occur in a later time window: within 4–20 years after diagnosis [3].

Several reports have described symptom changes in cases of Borden type conversion. Cognard et al. reported the details of seven patients who displayed conversion to a higher type of Borden classification; they found concomitant symptom worsening in all seven patients [7]. In the study by Kim et al., symptom worsening (including appearance of new symptoms) was observed in three of the four patients with conversion to a higher type of Borden classification [6]. Shah et al. observed symptom worsening (including appearance of new symptoms) in three of the four patients with Borden type changes; on the other hand, 4 of the 21 patients who did not display type conversion also showed exacerbation of symptoms [3]. These studies suggest that symptom exacerbations may have a high sensitivity, but low specificity for a conversion to a higher type of Borden classification. In our study, too, none of the six patients with symptom exacerbations showed a type change.

The follow-up assessment of this prospective study was primarily based on clinical evaluation and MRI with specific interest in time-of-flight MRA and T2-star/susceptibility-weighted images. The sensitivity and specificity of time-of-flight MRA for the diagnosis of DAVF is reported to be relatively high: sensitivity 64–91% and specificity 80–98% [13–15]. On the other hand, the accuracy of RLVD detection with time-of-flight MRA is slightly inferior: sensitivity 58–83% and specificity 77–93% [16]. In addition, although reports on metrics like sensitivity and specificity are few, a T2-star or susceptibility-weighted image is also reported to be useful in detecting cerebral venous congestion [17, 18]. These imaging assessments with MRI are not perfect for detecting the type conversion of DAVF, and the question of when to move to more invasive, but the gold standard testing (angiography) is a difficult problem. As discussed earlier, if stenotic changes are already present in the affected sinus at initial diagnosis, the chance of dynamic change in the angioarchitecture in the future is higher. Although such changes in angioarchitecture lead to shunt flow reduction or obliteration in our cases, occlusive change in the draining dural sinus might lead to a worsening of Borden classification. We suggest that more careful follow-up is necessary for Borden type I patients with stenotic or occlusive changes in the affected sinus at initial diagnosis. As neuroimaging changes have been observed even at the timing of 3-year follow-up, follow-up for > 3 years is suggested for patients.

There were several limitations to this study. First, the sample size was relatively limited according to the rarity of the disease and was subject to a certain chance of type II error. Second, 17% of patients dropped out during follow-up, which could influence the reliability of the results. Third, 34% of the cases received interventions, which led to difficulty in analyzing the pure natural history of the disease. Finally, since routine angiography during the follow-up period was not planned in this study, the follow-up assessment of shunt flow was based on MRI, which led to uncertainty for the precise evaluation. Otherwise, the periodic neuroimaging data of the entire population would offer some useful information on the clinical course of the disease.

## Conclusions

We reported the clinical outcomes of patients with Borden type I intracranial DAVF who were prospectively registered to the study. Borden type I DAVF displayed a benign clinical course; none of these patients experienced conversion to a higher type of Borden classification or intracranial hemorrhage. While both clinical symptoms and neuroimaging findings are stable in 70–75% of the patients, more than 10% of the patients displayed a tendency toward symptom improvement, and 7% of the patients displayed spontaneous shunt flow disappearance on MRA. On the other hand, 21% of the patients had worsening symptoms and 35% required palliative treatment.

According to the subgroup analysis, the restrictive changes of draining sinuses at initial diagnosis might be an imaging biomarker for future shunt flow reduction and obliteration.
